# Addressing the Gender Gap in Residency Awards Using a Blinded Selection Process

**DOI:** 10.1212/NE9.0000000000200136

**Published:** 2024-06-12

**Authors:** Clare M. Lambert, Reshma Narula, Vanessa Cooper, Jeremy J. Moeller

**Affiliations:** From the Department of Neurology, Yale School of Medicine, New Haven, CT.

Despite a recent increase in the proportion of practicing neurologists who identify as women, there remains relatively few women neurologists who hold leadership positions and who receive prestigious academic awards or research grants.^1^ This Viewpoint article highlights gender bias in academic neurology and describes an intervention for mitigating bias in resident awards.

In 2022, 50% of assistant professors of neurology in the United States identified as women. This number dropped to 42% at an associate professor level and 26% at a professor level.^2^ Awards during residency training can foster career growth and progression toward leadership positions. Silver et al.^3^ highlighted that between 1955 and 2017, only 18% of American Academy of Neurology (AAN) award recipients identified as women based on published records of recipients of 20 different AAN award categories. A subanalysis of the latter 10 years of data (2008–2017) revealed only a 4% increase in women awardees. From 1948 to 2023 there have been only 3 woman presidents of the AAN, compared with 34 men.^4^ Currently, there is no lack of highly qualified women neurologists, with women comprising half the workforce at the assistant professor level, yet the demographics of award winners and leadership remain relatively unchanged. The gender gap seems to widen in congruence with the prestige of award and the level of leadership position. We must consider the possibility that unconscious gender bias plays a role in how winners are selected. To examine this, we implemented a blinded merit-based approach for selecting the recipient of a junior neurology resident award in clinical excellence at our institution. From 2003 to 2018, an award (the Lewis Levy Award) was presented to the postgraduate year (PGY)-2 resident deemed most deserving of acknowledgement and a travel bursary for an academic meeting. There were no specified criteria, and the winner was determined by faculty votes. Only clinical faculty voted. In 2019, the method of selecting the award winner was changed to a structured, blinded format whereby each resident was required to submit a case report that was deidentified and independently reviewed by 5 different faculty members, using a structured scoring rubric, focusing on the quality of case, clarity of communication, and appropriateness of figures and references. The award was given to 2 recipients each year. Only clinical case reports were accepted as this award aimed to acknowledge clinical excellence as opposed to excellence in other scholarly endeavors. The sex of each clinical faculty member, resident, and award recipient for each year was collected and tabulated, using data from program records and publicly available information. We compared the gender ratios of each group before and after the intervention, and a voter was counted each year they voted. A 2-sided Fisher exact test was used for statistical comparisons.

From 2003 to 2018, only 8% (4/52) of PGY-2 women residents won the award, compared with 20% (12/60) of men. From 2019 to 2023, 33% (10/30) of PGY-2 women residents won the award and 100% of award winners identified as women (*p* = 0.004), despite no statistically significant change in the gender ratio of PGY-2 residents (53.2% men before intervention vs 38.8% after, *p* = 0.122). In the postintervention period, 68% of faculty voters identified as men compared with 76% before (*p* = 0.002). The current case report judges consist of 3 men and 2 women since 2021 and 4 men and 1 woman from 2019 to 2020.

The observed change in gender composition of awardees raises the question: Is our perception of a “good neurologist” grounded in clinical competency or in familiarity? Perhaps we may relate more easily to those who look or act more like us, which could certainly help explain why awardees' demographics mimic that of voters. In addition, the award is named after a man, which could further deepen a preexisting bias.

Limitations include the fact that a deidentified case report is likely testing for a different type of clinical excellence than voting for the perceived “best” resident. In addition, this was an observational study, thus we obtained gender identity data from publicly available records, rather than self-identified data, similar to methods applied in previously published work.^3^ We could not track the voting patterns of individual faculty members, so it is not possible to confirm that men were more likely to vote for a resident who was a man or whether there was a more persistent bias regardless of faculty sex. Finally, all the faculty or residents included in this study identified as men or women, so generalizability to other populations may be limited in this regard. Exploring different ways to mitigate gender bias in academic neurology remains a priority and could further be supplemented through conscious sponsorship and nomination of excellent woman neurologists for awards and promotions. Overall, our experience suggests that a structured, blinded process may help to reduce bias and promote gender equity in neurology.

## Appendix Authors

**Table:**

Name	Location	Contribution
Clare M. Lambert, MD	Department of Neurology, Yale School of Medicine, New Haven, CT	Drafting/revision of the manuscript for content, including medical writing for content; major role in the acquisition of data; study concept or design; analysis or interpretation of data
Reshma Narula, MD	Department of Neurology, Yale School of Medicine, New Haven, CT	Drafting/revision of the manuscript for content, including medical writing for content; study concept or design
Vanessa Cooper, MD	Department of Neurology, Yale School of Medicine, New Haven, CT	Drafting/revision of the manuscript for content, including medical writing for content; study concept or design
Jeremy J. Moeller, MD, MSc, FRCP	Department of Neurology, Yale School of Medicine, New Haven, CT	Drafting/revision of the manuscript for content, including medical writing for content; major role in the acquisition of data; study concept or design; analysis or interpretation of data
